# Update on Autophagy Inhibitors in Cancer: Opening up to a Therapeutic Combination with Immune Checkpoint Inhibitors

**DOI:** 10.3390/cells12131702

**Published:** 2023-06-23

**Authors:** Eloïne Bestion, Eric Raymond, Soraya Mezouar, Philippe Halfon

**Affiliations:** 1 Genoscience Pharma, 13006 Marseille, France; eraymond@hpsj.fr (E.R.); s.mezouar@genosciencepharma.com (S.M.); phalfon@genosciencepharma.com (P.H.); 2 Department of Medical Oncology, Paris Saint-Joseph Hospital Group, 75014 Paris, France; 3 Établissement Français du Sang, Provence Alpes Côte d’Azur et Corse, Marseille, France; «Biologie des Groupes Sanguins», Aix Marseille Univ-CNRS-EFS-ADÉS, 13005 Marseille, France

**Keywords:** autophagy, cancer, drug inhibitor, PPT1, combinational therapy, clinical trial

## Abstract

Autophagy is a highly conserved and natural degradation process that helps maintain cell homeostasis through the elimination of old, worn, and defective cellular components, ensuring proper cell energy intake. The degradative pathway constitutes a protective barrier against diverse human diseases including cancer. Autophagy basal level has been reported to be completely dysregulated during the entire oncogenic process. Autophagy influences not only cancer initiation, development, and maintenance but also regulates cancer response to therapy. Currently, autophagy inhibitor candidates mainly target the early autophagy process without any successful preclinical/clinical development. Lessons learned from autophagy pharmaceutical manipulation as a curative option progressively help to improve drug design and to encounter new targets of interest. Combinatorial strategies with autophagy modulators are supported by abundant evidence, especially dealing with immune checkpoint inhibitors, for which encouraging preclinical results have been recently published. GNS561, a PPT1 inhibitor, is a promising autophagy modulator as it has started a phase 2 clinical trial in liver cancer indication, combined with atezolizumab and bevacizumab, an assessment without precedent in the field. This approach paves a new road, leading to the resurgence of anticancer autophagy inhibitors as an attractive therapeutic target in cancer.

## 1. Introduction

Autophagy is subdivided into three main types: macroautophagy, microautophagy, and chaperone-mediated autophagy. All of them work on the removal of unnecessary or dysfunctional cellular components, ensuring the maintenance of cellular homeostasis and survival [1,2]. In this review, macroautophagy, hereafter referred to as autophagy, is discussed.

**Figure 1:**
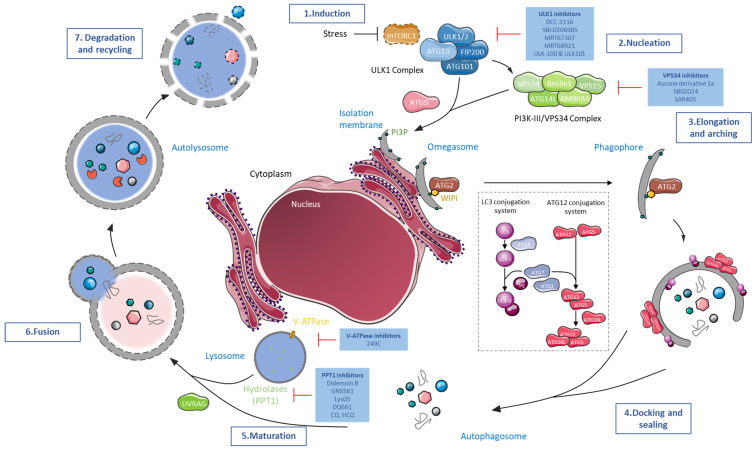
The molecular machinery of mammalian canonical autophagy and its pre-clinical and clinical targets. The complex autophagy process can be dissected into seven steps. (1) The induction phase of autophagy is controlled by the Unc-51-like autophagy activating kinase 1 complex (ULK1 complex), composed of ULK1/2 proteins and three other protein partners: the FAK family-interacting protein of 200 kD (FIP200), autophagy-related protein 13 (ATG13), and autophagy-related protein 101 (ATG101), to mediate the mammalian/mechanistic target of rapamycin (mTOR) signals. The ULK1 complex assimilates the stress signal received from mTOR complex 1 (mTORC1), activates it, and is responsible for the initiation of phagophore formation, the unclosed autophagosome structure. Immediately downstream of ULK1 complex activation, the class-III phosphatidylinositol 3-kinase (PI3K-III)/vacuolar protein sorting 34 (VPS34) complex, formed by VPS34 and by regulatory proteins vacuolar sorting protein 15 (VPS15), Beclin1 (BECN1), autophagy and beclin 1 regulator 1 (AMBRA1) and ATG14L, is phosphorylated and activated. This new complex participates in phagophore (2) nucleation and membrane isolation, elongation, and completion. It furthermore controls the conversion of phosphatidylinositol (PI) into phosphatidylinositol 3-phosphate (PI3P). Local PI3P production occurs at a characteristic ER structure called the omegasome. PI3P then recruits the PI3P effector proteins WD repeat domain phosphoinositide-interacting proteins (WIPI) to the omegasome via interaction with their PI3P-binding domains. (3) Follows the preparation of phagophore membranes for phagophore recruitment to conjugation systems responsible for autophagosome formation. The two ubiquitin-like conjugation systems include the Atg12 conjugation system and the microtubule-associated protein 1A/1B-light chain 3 (LC3) conjugation system, both catalyzed by ATG7. The Atg12 conjugation system forms after ATG12 conjugates to ATG5, both stabilized by the ATG16L protein. The recruitment of the ATG12-ATG5–ATG16L1 complex starts provoking ATG3-mediated conjugation, crucial for the two conjugation systems’ end of execution. In the meantime, the LC3 conjugation machinery has conjugated microtubule-associated protein light chain 3 (LC3) with ATG4 forming LC3-I which is then converted into LC3-II after LC3-I conjugation with membrane-resident phosphatidylethanolamine (PE). The attached-LC3-II to the autophagosome serves as a docking site for the ubiquitin-binding protein (p62) and neighbor of the BRCA1 gene 1 protein (NBR1) that will trap organelles and proteins tagged by ubiquitination for their autophagic degradation. (4) Full execution of the conjugation systems and the presence of associated-LC3-II with PE on the forming autophagosome will participate in its elongation, arching, and closing. Sealing of the phagophore membranes gives rise to a double-layered vesicle called the autophagosome. (5) The autophagosome vesicle then matures before its (6) fusion with the lysosome, forming an autolysosome degradative structure. (7) Acidic lysosomal hydrolases are released nearby the previous embedded cellular components leading to their breakdown into essential products before being discharged and used for the biosynthesis of new components and to fuel cells. Multiple early (ULK and VPS34 inhibitors) and late (V-ATPase and PPT1 inhibitors) pharmacological autophagy blockers are currently under pre-clinical, and for some, clinical evaluation.

The autophagy mechanism, active at the basal level in most healthy cell types [3,4], is a highly regulated pathway accompanied by tens of autophagy-related (ATG) genes encoding for crucial autophagy proteins [5,6]. Some of which, referred to as the ‘core’ molecular machinery, are particularly essential for autophagy-related compartments formation. The first central autophagy body is the autophagosome, a double-membrane structure able to sequester cellular components that will thereafter merge with the second primordial autophagy body, the lysosome. This acidic vesicle encompasses a variety of hydrolases able to breakdown embedded cellular components into essential products. They are thereafter discharged into the cytoplasm and used for the biosynthesis of new components, enabling cell sustainability. As a crucial survival mechanism, the autophagy molecular pathway has been extensively studied (Figure 1).

**Figure 2:**
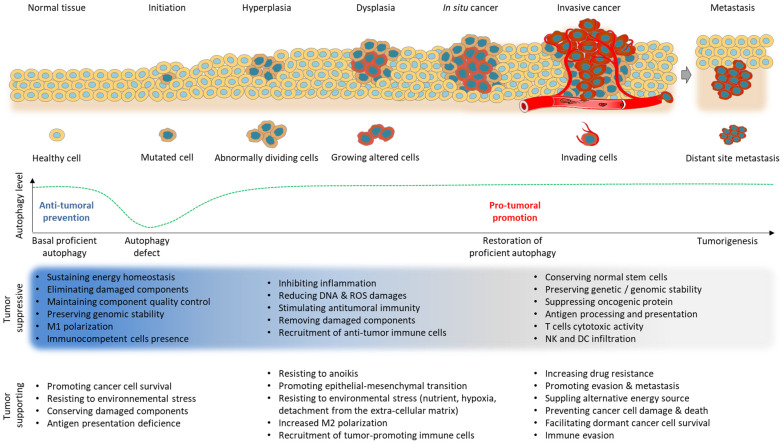
Autophagy—a double-edged-word in cancer. Tumorigenesis starts with an oncogene mutation in the healthy tissue that makes cells grow and divide in an uncontrolled fashion, rapidly evolving from the hyperplasia-to-dysplasia stage followed by the in situ cancer stage. The altered cells exhibit changed morphology and behavior while they display an immature phenotype. Tumor cells then invade close-by areas until lymphatic and blood circulating system spread from the primary tumor site to distant sites, forming metastasis. Autophagy’s highly dynamic mechanism is permanently modulated during the tumorigenic process, working for both cancer suppression and promotion.

Autophagy deregulation is associated with various human pathogeneses such as cardiovascular diseases, immune diseases, and cancers [5,7,8,9,10]. Autophagy baseline level fluctuations were first described in cancer cells in the late 1970s [11,12], then demonstrated to be observable during the entire oncogenic process [13,14] (Figure 2). Paradoxical roles of autophagy in tumor suppression and tumor promotion rapidly surfaced, depending on not only the autophagy basal level but the tumor type, disease stage, and genetic background as well [6,15,16]. It is indeed widely admitted that autophagy behaves as a tumor-suppressor mechanism at the very early stages of tumorigenesis [13,17], with its deficiency enabling tumor formation. Conversely, once a tumor has formed and grows, proficient autophagy instead provides assistance to cancer cells to respond to various environmental stresses such as nutrient deprivation, hypoxia, metabolic stress, and detachment from the extracellular matrix (ECM), ensuring their survival and the formation of metastatic sites [16,18,19,20,21,22]. It is thus commonly admitted that for most advanced cancers, the autophagy level is promulgated, promoting cancer progression. Autophagy disruption has been either connected to core ATG genes alteration, including BECN1, ATG1, ATG5, ATG7, ULK1, or Atg16L1 [4,23,24,25,26,27], and, more recently, the lysosomal enzyme palmitoyl-protein thioesterase 1 (PPT1) [28,29,30].

Autophagy machinery has therefore turned out to be an attractive therapeutic target in cancer, especially for dealing with process inhibition when it comes to advanced stages. Recently, an autophagy combinatorial strategy with immunotherapies has been put forward since studies have demonstrated that the use of autophagy inhibitors triggers immune checkpoint inhibitors (ICIs) efficacy, thus improving anticancer response [31,32,33]. Also, pharmacologic autophagy inhibition participates in immune response recovery, with it being an attractive approach to fight cancer therapeutic resistance [30,34].

A review of the latest and major small molecules able to inhibit the autophagy mechanism has been realised in regard to their mode of action and both their preclinical and clinical results. Recent advances dealing with the relevance of the autophagy inhibitor combination strategy are as well examined, focusing on the use of immune checkpoint inhibitors. We have further discussed the new identified autophagy targets for drug development, opening up on targeted protein degradation strategies.

## 2. Targeting Cancer with Autophagy Inhibitors

A diverse range of autophagy inhibitors have been developed and tested throughout the last few decades. Most of them turned out to be key tools for the mechanistic study of the autophagy process and for novel therapeutic agents’ design since they displayed significant side effects, thus obstructing their application in clinical trials [35,36,37]. A few autophagy inhibitors are currently under the spotlight as cancer drug candidates. Hereafter, the main and latest tested inhibitory molecules according to their targeted autophagy-related factors are reported.

### 2.1. ULK1/2 Inhibitors

The Unc-51-like kinase 1 (ULK1) complex consists of ULK1 and ULK2 proteins and the protein partners ATG13, ATG101, and FIP200 to mediate MTOR signals. Because the complex is required during the early steps of autophagosome biogenesis [38,39,40], the developed ULK1/2 inhibitors block early autophagy mainly through the inhibition of the ULKs’ kinase activity. Some of them compete with the ATP binding site, including MRT67307 and MRT68921, an improved MRT67307-derivative compound [41]. Both of the last two reversible kinase inhibitors were found to specifically block the function of TANK-binding kinase 1 (TBK1) [41,42]. They are able to disrupt autophagosome maturation in mouse embryonic fibroblasts (MEFs), blocking autophagic flux [43].

SBI-0206965, a highly specific inhibitor of ULK1 developed from a focal adhesion kinase (FAK) inhibitor, suppresses ULK1-mediated phosphorylation events in cells [44]. The compound has displayed tumor growth inhibition properties both in vitro and in vivo in various cancer types via dual autophagy and apoptosis pathways [44,45,46,47]. SBI-0206965 is also a direct inhibitor of AMP-activated protein kinase (AMPK) that activates not only the ULK complex [48] but that of FAK as well [44], making its specificity disputable.

More recently, Martin et al. reported ULK-100 and ULK-101 small molecules and demonstrated their in vitro superior potency and selectivity over SBI-0206965 [49]. The molecules were able to sensitize KRAS mutant lung cancer cells to nutrient stress, and ULK-101 allowed the inhibition of both the nucleation of autophagic vesicles and turnover.

DCC-3116 was demonstrated to be a potent and selective dual inhibitor of ULK1 and ULK2, acting at the nanomolar range [50]. The small molecule was further outlined to block autophagosome formation and lysosomal degradation. The compound is particularly attractive in multiple RAS cancer cell lines including lung, pancreatic, colorectal (CRC), and melanoma cancer. Interestingly, DCC-3116 was shown to inhibit compensatory autophagy from KRAS G12C inhibitors in vitro [51]. DCC-3116, as monotherapy and in combination with trametinib, binimetinib, or sotorasib, is currently under phase 1/2 clinical trials in patients with advanced or metastatic solid tumors exhibiting an RAS/mitogen-activated protein kinase (MAPK) pathway mutation (NCT04892017).

### 2.2. Vacuolar Protein Sorting 34 (VPS34) Inhibitors

Pasquier et al. worked on the discovery of new specific VPS34 kinase inhibitors identifying a series of tetrahydropyrimidopyrimidinone derivatives [52], starting with hit compound 1a to compound 31 after medicinal chemistry optimization. Some of them showed potent antiproliferative and apoptotic effects, both in vitro and in vivo in various cancer types [53,54,55,56].

Recently, aurone derivative 1a, an ATP-competitive inhibitor of the VPS34 inhibitor, has been demonstrated to suppress autophagy in cell-based assays [57] and in in vivo models [58].

Considering breast cancer, SB02024 blocks autophagy in vitro, reduces xenograft growth in two breast cancer cell lines in vivo, and significantly improves sensitivity to sunitinib and erlotinib [59]. Noman et al.’s study revealed, using SB02024 or SAR405, a decrease in tumor growth and improved mice survival in melanoma and CRC tumors [60]. Noman laboratory also revealed that targeting VPS34 in animal models turns cold tumors into hot, inflamed tumors in melanoma and CRC, thus enhancing the efficacy of anti-programmed death-ligand 1 (PD-L1)/programmed cell death-1 (PD-1) blockade [60]. The combinatorial strategy indeed enhances the infiltration of CD8+ T cells and M1 macrophages into the tumor site. SAR405 additionally hampers autophagy and synergizes with MTOR inhibition in renal tumor cells [61]. The principal issue with the use of VPS34 inhibitors is their implication in non-autophagic late endocytic trafficking [62], compromising drug specificity. To date, none of the abovementioned inhibitors has entered clinical trials.

### 2.3. V-ATPase Inhibitors

Vacuolar-ATPase (V-ATPase) is a proton pump responsible for cellular homeostasis. The multi-subunit transmembrane complex controls, among others, lysosome acidification, indirectly regulating autophagic flux [63]. The most commonly employed vacuolar-H+ ATPase inhibitor encompasses pepstatin A, leupeptin, E64d, salinomycin, and bafilomycin which thereby alkalinize lysosomal compartment, blocking autophagosome–lysosome fusion [64]. Those V-ATPase inhibitors, however, display tolerability and pharmacological issues limiting their use to laboratory tools.

Recently, a panel of Ras-mutant preclinical cancers models was found to be sensible to the 249C cytotoxic agent with nanomolar potency [65]. V-ATPase subunit ATP6V1H was pointed out as a 249C drug target, provoking inhibition of its biochemical activity, lysosomal acidification, and macropinocytosis.

### 2.4. PPT1 Inhibitors

PPT1 is an enzyme responsible for protein depalmitoylation, regulating autophagy. PPT1 is a mobile depalmitoylating enzyme responsible for the cleavage of thioester linkages in palmitoylated proteins, facilitating their degradation. The hydrolase was identified to account for the proper localization of V-ATPase components onto the lysosomal membrane [66,67]. Indeed, chemical PPT1 inhibition induces V-ATPase subunit V0a1 misrouting, which dysregulates lysosomal acidification and thereby the autophagy process. PPT1 expression is high in many cancer types [68]. PPT1-dependent depalmitoylation regulates intracellular trafficking of a considerable number of proteins including most of the autophagy [69] and MTOR proteins [70]. Therefore, the development of potent PPT1 inhibitors is of strong interest in cancer.

Didemnin B, a cyclic depsipeptide, exhibits a potent antiproliferative effect and immunosuppressive activity both in vitro and in vivo against a panel of human cancers at low dosages [71]. It was later found to selectively elicit massive apoptosis via the dual inhibition of eukaryotic translation elongation factor 1 alpha 1 (EEF1A1) and PPT1 [72]. The marine natural product was the first to enter clinical development in the U.S. in cancer indication [73,74]. A phase 1 study led in Europe revealed that muscle toxicity was dose-limiting while the addition of carnitine allowed dosage escalation [75]. In 2018, Didemnin B obtained marketing authorization approval in the Asia–Pacific region for the treatment of multiple myeloma [76].

Chloroquine (CQ) and hydroxychloroquine (HCQ) antimalaria compounds are preferentially localized in lysosomal compartments due to their physicochemical properties [77,78]. Both drugs then provoke lysosomal compartment deacidification, stopping lysosomal enzyme activity, leading to autophagic flux inhibition and disruption of autophagosome–lysosome fusion [79]. HCQ was developed in response to the unexpected adverse effects of CQ treatment, such as severe constipation, gastrointestinal toxicity, cardiomyopathy, grade 3 fatigue, anemia, or retinal toxicity, all monitored in various cancer types [80,81,82,83]. HCQ anticancer drug effect evaluation during clinical trials revealed mixed results, exhibiting a partial [80,84,85] or insufficient antitumoral response compared to the standard of care [86,87,88]. Both CQ and HCQ autophagy inhibitors have nevertheless reached phase 4 clinical trials whilst never obtaining market approval for cancer indication. Nevertheless, their extensive investigation paves the road to the design and development of new potent autophagy inhibitors. 

Amaravadi worked on Lys05, a bivalent form of HCQ [89], with it having more potent antitumor activity both in vitro and in vivo than HCQ as a single agent in multiple human cancer cell lines and xenograft models [90]. Following the idea of autophagy and MTORC1 regulators, they also studied DQ661. The group published two papers where they identified PPT1 as a target of DQ661, HCQ, and Lys05, while the molecular target of these drugs was unknown at that point [28,67]. 

GNS561, a lysosomotropic small molecule that modulates the autophagy process presents strong anticancer activity in a wide panel of cancers with preferential accumulation and efficacy in the liver [29]. Once located in lysosomal compartments, the molecule alters the structural and functional integrity of lysosomes, blocking the late stage of the autophagy process. The compound binds to PPT1 and reduces not only its enzymatic activity but its expression as well. GNS561 successfully completed a phase 1b trial in patients with primary and secondary liver cancer, highlighting the safety of the molecule [91]. GNS561 is now entering a phase 2 clinical trial in association with standard-of-care atezolizumab–bevacizumab as a first-line treatment in patients suffering from unresectable hepatocellular carcinoma (NCT05448677) (Table 1).

## 3. Rational of the Combination of Autophagy Inhibitors with Immune Checkpoint Inhibitors

### 3.1. The Immune System and Autophagy

**Figure 3:**
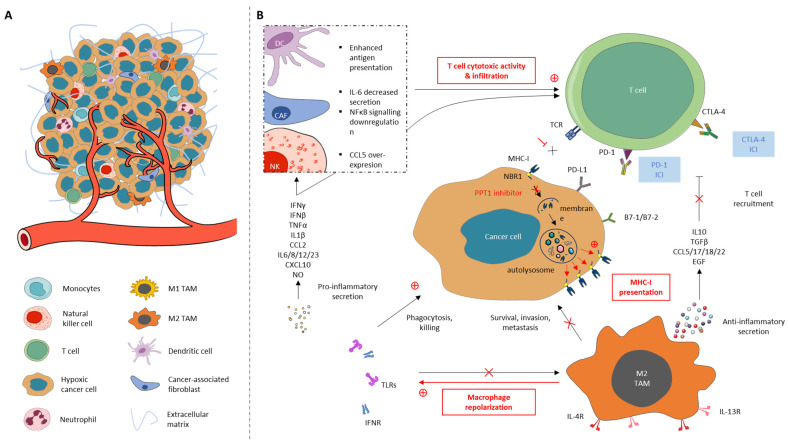
(**A**) The main cellular components of the tumor microenvironment (TME) and (**B**) immune cells’ cross-talk with cancer cells associated with therapeutic strategies. (**A**) The TME encompasses immune cells, stromal cells, the extracellular matrix (ECM), and blood vessels. It is composed of cancer and heterogeneous non-malignant cells integrated into a complex molecules and matrix network. The relevant cellular components are T cells including cytotoxic T cells (CD8^+^), tumor-associated macrophages (TAMs), natural killer (NK) cells, cancer-associated fibroblasts (CAFs), dendritic cells (DCs), neutrophils, and monocytes. (**B**) MHC-I degradation through the autophagy process using the NBR1 cargo protein prevents TCR recognition leading to T cells’ anti-tumoral cytotoxic activity blockade. PPT1 inhibitor use allows for increased MHC-I molecule presentation on cancer cells’ surface, restoring T cells’ killing capacity towards malignant cells. PPT1 inhibitor treatment further leads to TAMs repolarization from the M2 to M1 phenotype, provoking pro-inflammatory secretion that boost NK cells’ and DCs’ infiltration into the tumor site, enabling T cells’ cytotoxic activity and infiltration. M1 abundance achieves cancer cell phagocytosis and killing, while the M2 phenotype’s low presence refrains not only cancer cells’ survival, invasion, and metastasis but increases T cells recruitment as well. PPT1 inhibitor use enhances immune checkpoint blockers’ efficacy, further dampening immune evasion.

Tumor cells continually interplay with their microenvironment which consists of cellular and non-cellular components [92] (Figure 3). The tumor microenvironment (TME) is of critical importance in tumor initiation, progression, metastasis, and resistance to treatment [93]. It further plays a significant role in tumor immune surveillance and immunological evasion [94,95,96,97]. Immune cells within the TME, encompassing CD8+ and CD4+ T cells, regulatory T lymphocytes, B cells, neutrophils, tumor-associated macrophages (TAMs), natural killer (NK) cells, and dendritic (DC) cells, decisively take part in this process.

As malignant cells, they display a diversity of relevant markers on their surfaces along with immune checkpoint molecules, essential for self-tolerance and immune response, enabling tumor suppression. However, interactions between some cancer cell-surface proteins with specific checkpoint proteins expressed on immune cells allow evasion from immune system anti-tumoral offensive [98,99]. Immune elimination failure mainly occurs through the disruption of two processes: (1) interaction between the major histocompatibility complex (MHC) on the surface of antigen-presenting cells (APCs), such as cancer cells and the T cell receptor (TCR), and (2) signalization displayed by antigen-independent co-signalling molecules, intimately regulated by co-stimulators or co-inhibitors known as immune checkpoints [100,101,102]. Downregulation of major histocompatibility complex class I (MHC-I) presentation has been observed in many different types of cancers [103,104,105,106,107] and is associated with poor prognosis. In addition, recognition of cytotoxic T-lymphocyte-associated protein 4 (CTLA-4) and PD-1 co-inhibitory receptors, found on the surface of cytotoxic T cells (CTLs), with their tumor cell surface partner proteins B7-1/B7-2 and PD-L1, respectively, causes a lack of suitable T cell priming and activation, putting brakes on unrestricted cytotoxic T effector function against malignant cells [108,109,110].

ICIs, able to hamper the ‘off’ signal between cancer cells and T cells, have rapidly surfaced as a promising therapeutic option for cancer patients in the recent decades and have consequently been developed [111,112,113,114,115]. The ones targeting CTLA-4 (ipilimumab), PD-1 (pembrolizumab, nivolumab, and cemiplimab), and its partner protein PD-L1 (atezolizumab, avelumab, durvalumab), are currently approved and used in more than 50 cancer types, administered either alone or in combination with chemotherapies or even between them, as first or second lines [116,117]. 

There is growing evidence showing that autophagy signalling coordinates the immune response [118,119,120,121]. Working on a pancreatic cancer model, a paper demonstrated that autophagy participates in immune evasion through the selective targeting of MHC-I molecules for degradation [34]. Molecules are selectively addressed in an autophagy-dependent mechanism experiencing lysosomal degradation through the NBR1 autophagy cargo receptor. Decreased presentation of the molecules on cancer cells’ surface leads to obstructed antigen presentation and T cells’ cytotoxic activity. Conditional whole-body Atg7-deficient mice implanted with liver cancer with a high tumor mutational burden in a study pointed out reduced intratumoral T cell exhaustion [122]. Deficiency of HIP1R in tumor cells was demonstrated to cause PD-L1 accumulation and suppressed T-cell-mediated cytotoxicity [123]. HIP1R actually targets PD-L1 for lysosomal degradation, inhibiting tumor growth by increasing T cell cytotoxicity. The results suggest that selective autophagic degradation of PD-L1 avoids cancer immune escape. However, PD-L1 palmitoylation decreases its endosomal-sorting-mediated autophagic degradation, highlighting PPT1 as an interesting autophagy-related target to control immune evasion [124]. In addition, Amaravadi Laboratory observed clear enhancement of T cell priming with PPT1 inhibition but not with genetic inhibition of upstream autophagy genes [30]. They further showed that PPT1 inhibition leads to IFN-β release from macrophages, essential for T-cell-mediated killing and conducted M2 to M1 phenotype switching in macrophages. The autophagy inhibitor CQ was also established to be able to reset TAMs from M2 to the tumor-killing M1 phenotype modulating antitumor immune response [125]. Autophagy thus promotes tumor growth by inhibiting T cell immune response. In addition, there is evidence that autophagy inhibition in NK and DC promotes their functions in the TME, increasing infiltration and cytotoxicity [126,127]. Proficient autophagy in cancer-associated fibroblasts (CAFs) was moreover shown to participate in tumor growth [127] and boost metastasis via interleukin-6 secretion and NFκB signalling [128]. In brief, autophagy inhibition could impact antigen presentation and phenotype repolarization, promoting immune cells’ anticancer response. Therefore, the therapeutic potential of autophagy modulators for controlling immunity has recently been considered.

### 3.2. Autophagy Combinatorial Strategy with Immune Modulators

Despite ICIs’ massive and rapid approval in cancer indication due to their improved risk:benefit ratio over the former standard-of-care treatment, it is worthy of note that the already completed clinical trials were more or less successful, sometimes exhibiting moderate or disappointing clinical results regarding certain types and subtypes of cancer when ICIs are administered in monotherapy [129,130,131,132,133]. Patient response rates rarely reached and exceeded 40%, which is not only negligible but insufficient [134]. ICI combination itself was further shown to expose the exacerbated severity of immune-related adverse events in some cancer patients [135]. Because accumulating evidence suggests that a lower proportion of cancer patients benefit from conducive ICIs’ anti-tumoral effect than predicted, a combinatorial strategy with autophagy inhibitors in order to reinforce and optimize their efficacy is being presently studied.

Non-small-cell lung cancer (NSLC) patients with inactivating mutations in liver kinase B1 (LKB1) show poor response to anti-PD-1 immunotherapy [136]. Interestingly, Deng et al. recently demonstrated that LKB1 loss suppresses antigen processing and presentation to MHC because of the increased autophagic flux and compromised proteasomal degradation activity of antigenic peptides. ULK1 inhibition (MRT68921) or lysosomal function impairment (CQ) blocked the autophagic degradation of immunoproteasome constituents, successfully restoring antigen presentation and leading to increased T cell infiltration and improving the response to anti-PD-1 treatment in an LKB1 mutant NSCLC mouse model. The Yamamoto team demonstrated in vitro that tumor-specific autophagy inhibition using CQ drives increased MHC antigen presentation in a pancreatic ductal adenocarcinoma (PDAC) model, enhancing CD8+ T cell proliferation, activation, and tumor cell killing [34]. They first pointed out that CQ monotherapy failed to significantly lower tumor weight or enhance T cell infiltration in mice bearing orthotopic PDAC tumor while working with in combination with anti-PD-1 and anti-CTLA4; synergistic and potent anti-tumor activity as well as an increased anti-tumor immune response were registered. Amaravadi et al. reported that HCQ in combination with anti-PD-1 resulted in tumor growth attenuation and raised survival in melanoma mouse models [30]. Chemical PPT1 inhibition led to macrophages’ repolarization from M2 to the M1 phenotype and augmented T-cell-mediated cytotoxicity. They further observed a significant diminution in myeloid-derived suppressor cell infiltration in the TME. PPT1-deficient DCs were observed to enhance the priming of naive CD8+ T cells during viral immune response, suggesting enhanced DC function in the TME following autophagy inhibition [127]. Recently, the GNS561 PPT1 inhibitor combined with anti-PD-1 was highlighted as an attractive approach to restore immune response in a transgenic immunocompetent hepatocarcinoma (HCC) mouse model [137]. MHC-I cancer cells surface expression increased and led to cytotoxic T cells’ recolonization in the tumor site. A phase 2 study evaluating GNS561 combination with atezolizumab plus bevacizumab is currently recruiting for the first-line treatment of unresectable HCC patients (NCT05448677), with it being the first trial to assess the benefits of the autophagy PPT1 inhibitor with immune therapy. Recent studies have therefore pointed out that autophagy inhibitors combined with ICIs could work together to not only significantly reduce cytotoxic T cell exhaustion but also reset TAMs from M2 to the M1 phenotype, with them being an attractive therapeutic strategy to fight cancer. Autophagy inhibitors may thus partially rub out ICIs’ lack of effectiveness and resistance, improving antigen presentation and resetting the anti-tumoral immune cell profile. However, this novel combination regimen needs to be further assessed in preclinical studies and, more importantly, needs to be followed by clinical evaluation.

## 4. Concluding Remarks and Future Perspectives

Targeting the autophagy process to fight cancer is a promising strategy, especially in the framework of combination. A broad range of developed autophagy inhibitors have shown encouraging preclinical results with no or low toxicity issues and with powerful anticancerous activity in the nanomolar/micromolar range. Clinical translations have nevertheless been unsuccessful, demonstrating worrisome adverse events and insufficient efficacy. Indeed, almost none of the evaluated compounds were initially designed to precisely target autophagy before being investigated in cancer indication. Some fresh, more potent and specific candidates have entered recently preclinical and clinical research programs with results expected in the upcoming years. Recent publications emphasise their potency in combination with single or dual ICI therapy since their synergistic activity is able to remobilize the cytotoxic effect of CTLs and repolarize TAMs. Currently, only the new GNS561 autophagy inhibitor in combination with atezolizumab plus bevacizumab has been evaluated in a phase 2 cancer clinical trial. MEKi, such as ICIs, have shown encouraging preclinical results, but they curiously provided insufficient-to-no clinical benefits [138]. Inhibition of KRAS-RAF-MEK-ERK signalling displayed autophagy promotion, protecting cancer cells from the cytotoxic effects of KRAS pathway inhibition [33]. Interestingly, DCC-3116 was found to inhibit MAPK inhibitor-mediated compensatory increased autophagy and to enhance the anti-tumoral activity of trametinib in vitro [50]. DCC-3116, as monotherapy and in combination with trametinib, binimetinib, or sotorasib, is currently under phase 1/2 clinical trials in patients with advanced or metastatic solid tumors exhibiting MAPK pathway mutation (NCT04892017). Although targeting autophagy in cancer patients is a former idea and its achievement is still at a very early stage, combination with targeted therapy and MEKi is of major interest.

A recent examination of the literature spotlights possible new autophagy regulators as targets for new drug development. Comprehension of autophagosome biogenesis has taken a step forward since TMEM41B-deficiency was identified as being responsible for paralyzed autophagy initiation and lipid mobilization [139,140]. ATG9A and ATG2A have also recently been revealed to be crucial for autophagosome formation during the elongation period [141]. The phosphorylation status of VTI1B by PTPN9 [142] and that of syntaxin 17 by TANK-binding kinase 1 (TBK1) [143] was reported to be decisive for not only the expansion of phagophore structures and early autophagosome formation but as well for assembly of the ULK1 complex controlling the formation of phagophore structures, respectively. Klionsky and colleagues recently presented evidence that ATG14 is not only necessary to ULK1 complex initiation but that is also regulates autophagosome–lysosome fusion [144]. Moreover, they provide key elements showing that autophagy-related protein isoforms can dissimilarly impact core autophagy machinery. Exploration of the targeted protein degradation pathway may also turn out to be a powerful tool to move forward unanswered questions that have until now dampened the development of novel autophagy inhibitors. Targeted protein degradation strategies started with the emergence of a class of small pharmacological agents called proteolysis-targeting chimeras (PROTACs) [145]. This technology enables one to examine targets that were previously considered undruggable and offers a reversible effect [146]. The technology further provides resilience to acquired mutations, enhanced selectivity, and lower dosing requirements [147]. Preclinical campaigns and early clinical development have been launched, with the first clinical proof-of-concept surfacing in 2020 against two well-established cancer targets, the oestrogen receptor (ER) (ARV-471) and the androgen receptor (AR) (ARV-110). PROTACs also have the potential to be first-in-class small-molecule drugs that target immune cell activation [148,149]. However, there is still some work to be carried out regarding PROTAC design as their high molecular weight potentially limits not only cell permeability but pharmacokinetics evaluations as well.

## Figures and Tables

**Table 1:** Latest developed and tested autophagy inhibitors in cancer indication. AMPK: AMP-activated protein kinase, CQ: chloroquine, HCQ: hydroxychloroquine, N/A: not applicable, not available, NCT: national clinical trial, PPT1: palmitoyl-protein thioesterase 1, TBK1: TANK-binding kinase 1, ULK1/2: unc-51-like kinase 1/2, and V-ATPase: vacuolar ATPase.

Autophagy Stage	Compound	Molecular Target	Clinical Trial
Initiation	MRT67307/MRT68921	TBK1	N/A
	SBI-0206965	ULK1-mediated phosphorylation, AMPK	N/A
	ULK-100/ULK-101	ULK complex	N/A
	DCC-3116		Phase 1/2 (NCT04892017)
Nucleation	SB02024	VPS34	N/A
	SAR405		N/A
Autophagosome–lysosome fusion	249C	V-ATPase subunit ATP6V1H	Data
	Didemnin B	PPT1	N/A
	CQ/HCQ		Phase 4
	Lys05		N/A
	DQ661		N/A
	GNS561		Phase 2 (NCT05448677)

## Data Availability

Not applicable.
